# The Expansion of the Pulmonary Rib Cage during Breath Stacking Is Influenced by Age in Obese Women

**DOI:** 10.1371/journal.pone.0110959

**Published:** 2014-11-05

**Authors:** Jacqueline de Melo Barcelar, Andrea Aliverti, Catarina Rattes, Maria Eduarda Ximenes, Shirley Lima Campos, Daniella Cunha Brandão, Guilherme Fregonezi, Armèle Dornelas de Andrade

**Affiliations:** 1 Departamento de Fisioterapia, Universidade Federal de Pernambuco, Pernambuco, Brazil; 2 Dipartimento di Elettronica, Informazione e Bioingegneria, Politecnico di Milano, Milano, Italy; 3 Departamento de Fisioterapia, Universidade Federal do Rio Grande do Norte, Natal, Brazil; Research Center Borstel, Germany

## Abstract

**Objective:**

To analyze in obese women the acute effects of the breath stacking technique on thoraco-abdominal expansion.

**Design and Methods:**

Nineteen obese women (BMI≥30 kg/m^2^) were evaluated by anthropometry, spirometry and maximal respiratory muscle pressures and successively analyzed by Opto-Electronic Plethysmography and a Wright respirometer during quiet breathing and breath stacking maneuvers and compared with a group of 15 normal-weighted healthy women. The acute effects of the maneuvers were assessed in terms of total and compartmental chest wall volumes at baseline, end of the breath stacking maneuver and after the maneuver. Obese subjects were successively classified into two groups, accordingly to the response during the maneuver, group 1 = prevalent rib cage or group 2 = abdominal expansion.

**Results:**

Age was significantly lower in group 1 than group 2. When considering the two obese groups, FEV_1_ was lower and minute ventilation was higher only in group 2 compared to controls group. During breath stacking, inspiratory capacity was significant differences in obese subjects with a smaller expansion of the pulmonary rib cage and a greater expansion of the abdomen compared to controls and also between groups 1 and 2. A significant inverse linear relationship was found between age and inspiratory capacity of the pulmonary rib cage but not of the abdomen.

**Conclusions:**

In obese women the maximal expansion of the rib cage and abdomen is influenced by age and breath stacking maneuver could be a possible therapy for preventing respiratory complications.

## Introduction

Obesity even without associated diseases can induce changes in the respiratory system, impacting on strength and endurance of the respiratory muscles [1]–[3], pulmonary gas exchange [4], [5], lung volumes [6], [7] and exercise tolerance [8]. These alterations may be exacerbated by abdominal and thoracic surgery of the upper compartment and increase the incidence of postoperative pulmonary complications such as atelectasis and pulmonary infections which may persist for several days [9]–[11]. It has been reported that obese patients undergoing general anesthesia develop five times more atelectasis during 24 hours after extubation than non-obese patients [12].

In order to prevent or treat respiratory complications and improve lung function in obesity, several techniques are employed for lung expansion, such as intermittent positive pressure breathing, deep breathing exercises, incentive spirometry, however, in the literature, the evidence for efficacy techniques is still controversial [13]–[15].

The breath stacking technique, developed by Marini et al. [16], consists in using a mask equipped with a one way valve preventing either expiration or inspiration and therefore facilitating either lung expansion from Functional Residual Capacity (FRC) to Total Lung Capacity (TLC) or lung emptying from FRC to Residual Volume (RV). This technique was originally designed to estimate the subcomponents of vital capacity, namely inspiratory capacity (IC) and expiratory reserve volume (ERV), in poorly or not cooperative patients. Breath stacking proved to be able also to improve lung expansion [16], [17], and therefore it appears to be an interesting therapeutic tool for reversing atelectasis in obese patients, who show reduced FRC and ERV and hypoventilation of lung bases, due to the accumulation of fat in the chest and abdomen.

Recently, we have demonstrated that obese women are characterized not only by lung restriction, but also by an altered thoraco-abdominal pattern during spontaneous breathing [18]. In the present work we hypothesized that not only spontaneous breathing, but also the maximal expansion of the chest in the different thoraco-abdominal compartments is influenced by obesity. The aim was therefore to analyze the acute effect of breath stacking on thoraco-abdominal expansion and operational volumes during the maneuver.

## Methods

### Subjects

The sample was composed by 19 obese women and 15 normal-weighted age-matched healthy women used as controls. Obese subjects were selected sequentially by convenience from the Hospital of the Federal University of Pernambuco, while healthy controls were volunteers recruited at the Department of Physical Therapy at the Federal University of Pernambuco. Inclusion criteria were: age comprised between 18 and 60 years, body mass index (BMI) ≥30 kg/m^2^ for the obese and ranging between 18.5 and 24.9 kg/m^2^ for the control group. Women with chronic pulmonary or neuromuscular diseases, smokers and incapable of performing procedures were excluded. All measurements were performed in the Laboratory of Cardiopulmonary Physiotherapy and Physiology, Department of Physical Therapy, Federal University of Pernambuco.

### Ethics statement

The study was approved by the local Research Ethics Committee of the Hospital Agamenon Magalhães. Written informed consent was obtained from volunteers who agreed to take part in the research.

### Anthropometric measurements

Anthropometric measurements were initially assessed with volunteers in the orthostatic position, wearing light clothes and barefoot. A digital balance (Welmy W300, São Paulo, Brazil; capacity = 300 kg, resolution = 50 g), and a 2-meter-long metric tape measure was used to determine waist circumference (WC), i.e. the lowest measure between the last rib and iliac crest, and hip circumference (HC), i.e. the highest measure in the buttocks region. These data were also used to calculate body mass index (BMI, Kg/m^2^) and waist-to-hip ratio (WHR).

### Lung function and respiratory muscle pressure

Spirometry was performed using a portable spirometer, Micromedical Microloop MK8 (Kent, England). The following measures were assessed: forced expiratory volume in the first second (FEV_1_), forced vital capacity (FVC), and the ratio FEV_1_/FVC. During assessment, subjects sat erect, with both feet on the floor and arms unsupported, using a mouthpiece and noseclip. They performed at least three forced vital capacity maneuvers, with a two-minute interval between them, in accordance with reproducibility and acceptability criteria of the American Thoracic Society (ATS) [19], pulmonary function test [20] and the predicted values proposed for Brazilian subjects [21]. A digital manometer (MVD-300, Globalmed, São Paulo, Brazil) connected to a mouthpiece with a 2 mm orifice was used to maximal inspiratory pressure (MIP) and maximal expiratory pressure (MEP), evaluated at RV and TLC, respectively.

### Opto-Electronic Plethysmography (OEP)

**Figure 1:**
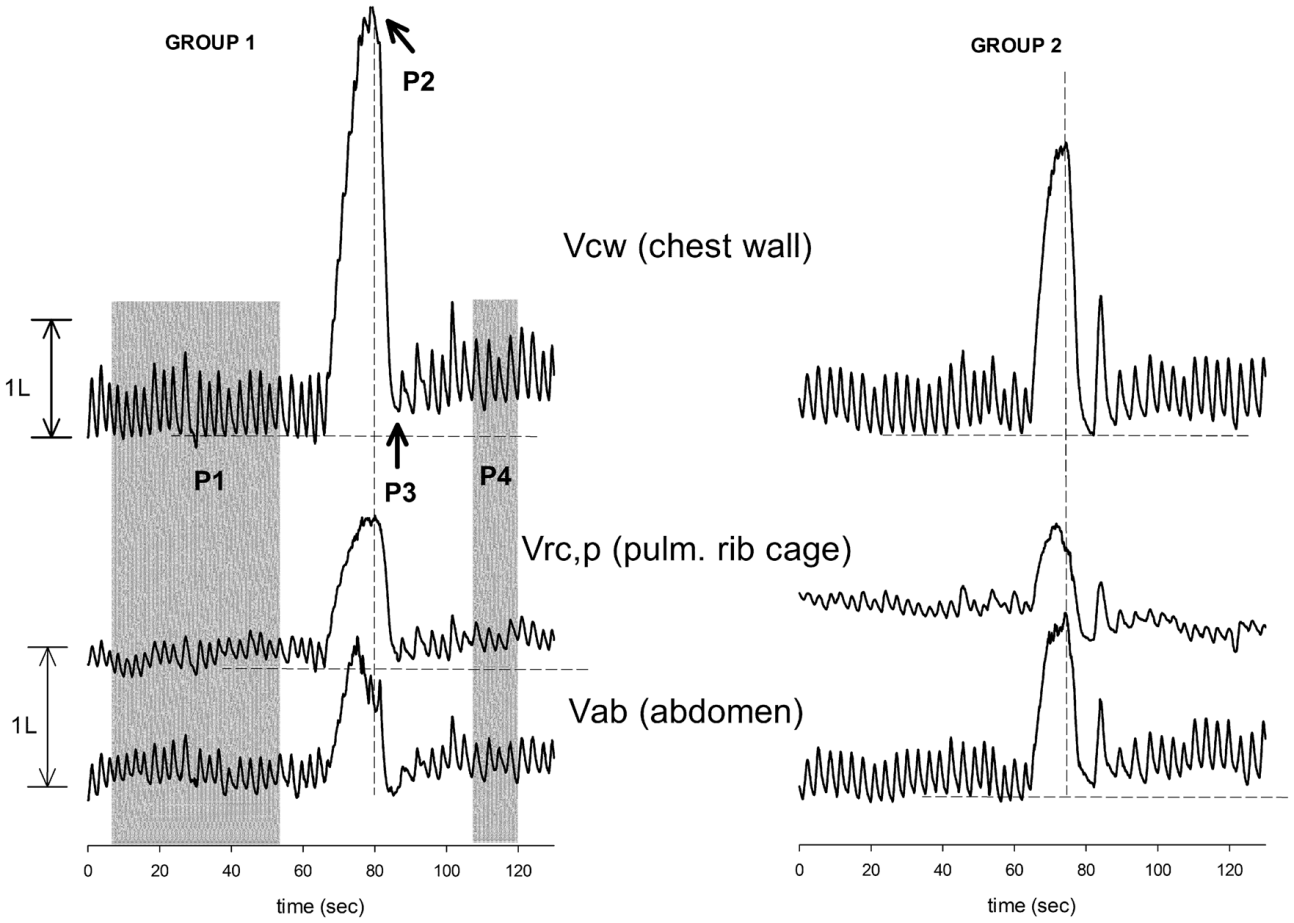
Representative tracings of total chest wall (top traces), pulmonary rib cage (middle traces) and abdominal (bottom traces) volume variations during the different phases of the test: spontaneous quiet breathing before the maneuver, breath stacking maneuver and recovery after the maneuver. P1: baseline period; P2: end of the breath stacking maneuver (Total Lung Capacity, TLC); P3: end of the first expiration after the breath stacking maneuver; P4: period after the maneuver. Tracings in control subjects, not shown, were generally similar to those recorded in group 1.

OEP (BTS Engineering, Milano, Italy) was used to assess thoraco-abdominal volumes and breathing pattern. According to the protocol, 89 reflective markers (diameter 5 or 10 mm) were used, fixed on the skin by hypoallergenic adhesive tape along seven horizontal rows (arranged circumferentially between the level of the clavicles and the anterior superior iliac spines) and five vertical columns (anteriorly and posteriorly), plus two additional columns in the midaxillary lines. Seven additional markers were placed to provide better detail in the anterior and posterior regions [22], [23]. OEP data were captured by eight cameras, four positioned anteriorly and four posteriorly with respect to the subject. After markers' positioning and a period of adaptation to the experimental conditions, all subjects were asked not to speak or move during the recording and analyzed while sitting on a rigid bed with both feet on the floor, knees and hips at about 90 degrees and the hands on the hips. OEP data were then recorded during a test composed by a) period of about 60 sec of spontaneous quiet breathing (QB) at rest, followed by b) a breath stacking maneuver and c) another period of QB during the recovery from the maneuver. During the breath stacking maneuver, a silicone face mask (Newmed, São Paulo, Brasil) was applied to the subject. A Wright MARK 8 respirometer (British Oxygen Company, London, England) attached to a connection on the mask allowed inspiration but not expiration. The other connection present on the mask was kept occluded. Before measurements, the subjects were instructed to voluntarily inspire during breath stacking, starting from FRC and gradually fill the lungs until reaching TLC over a period of about 20 seconds (Fig. 1). The termination of the maneuver was determined by observing no changes of inspired volume in the respirometer. After termination of the maneuver, the mask was promptly removed by the researcher, allowing the subject to freely inspire and expire again. In each subject, tests were repeated three times, with an interval of at least two minutes in between. For each test, the researcher took note of the inspired lung volume as measured by the Wright respirometer.

### Data analysis

From the three-dimensional coordinates of the markers, measured by OEP system, the volume enclosed by the entire chest wall (V_CW_) and its different thoraco-abdominal compartments (pulmonary rib cage- V_RC,P_, abdominal rib cage -V_RC,A_ and abdomen - V_AB_) was calculated as previously described [24], [25]. From variations of V_CW_ during quiet breathing the ventilatory pattern, in terms of minute ventilation, breathing frequency, inspiratory (T_I_) and expiratory (T_E_) time, tidal volume (V_T_) and percentage contribution of each compartment to tidal volume, was determined. The acute effects of the breath stacking maneuver were assessed in terms of V_CW_, V_RC,P_, V_RC,A_ and V_AB_ at baseline (BL), as average end-expiratory volumes of 4 stable breaths), end of the breath stacking maneuver, i.e. at TLC (end-BS), end of the first expiration after the breath stacking maneuver (post-1) and 30 seconds after the maneuver, as average end-expiratory volumes of 4 breaths (post-2) (Fig. 1). Inspiratory capacity (IC) of the total chest wall (IC_CW_), pulmonary rib cage (IC_RCp_), abdominal rib cage (IC_RCa_) and abdomen (IC_AB_) were calculated as the difference between total and compartmental volumes determined at end-BS and BL. For each subject, all data in the file with the best breath stacking maneuver (i.e., with the highest IC_CW_) was considered for data analysis.

Obese subjects were successively classified into two groups, accordingly to the response to the breath stacking maneuver. Subjects whose inspired volume during breath stacking was greater in the pulmonary rib cage than in the abdomen were classified as belonging to Group 1 (prevalent rib cage compartment). Vice versa, subjects whose inspired volume during breath stacking was greater in the abdomen than in the pulmonary rib cage were classified as belonging to Group 2 (prevalent abdominal compartment). Healthy subjects were classified as a single control group as no significant differences emerged in the response to the breath stacking maneuver.

### Statistical analysis

Statistical analysis was carried out using SPSS 18.0 software (Statistical Package for the Social Sciences). The Kolmogorov-Smirnov and Levene tests were used to verify sample normality and intergroup homogeneity. Group data were summarized using means and standard deviations. Differences between groups were evaluated by Mann Whitney test, Kruskal-Wallis test and Dunn's Multiple Comparison post hoc test. All tests were conducted at a 95% confidence level and significance level of p<0.05.

## Results

Anthropometric, lung function and respiratory muscle data are shown in Table 1. Age was significantly lower in group 1 than group 2 (p = 0.023), while no significant differences were found between the two groups of obese women in terms of anthropometric parameters (WC, HC, BMI and WHR). Among spirometric parameters only FEV_1_ was significantly lower in obese women. When considering the two obese groups, FEV_1_ was significantly lower than controls only in group 2 (p = 0.019). No significant differences were found in maximal inspiratory and expiratory pressures between groups.

**Table 1:** Anthropometric characteristics, lung function and respiratory muscle of control, obese (overall), group 1 and group 2.

	Control	Obese (overall)	Obese (Group 1)	Obese (Group 2)
**N. of subjects**	15	19	9	10
**Age (years)**	33.80±9.34	37.00±9.64	31.11±6.70	42.30±8.94^°^
**BMI (kg/m^2^)**	21.98±1.95	45.12±7.14***	43.45±9.41***	46.63±4.22***
**WC (cm)**	0.73±0.05	1.20±0.16***	1.14±.021***	1.24±0.10***
**HC (cm)**	1.00±0.05	1.36±0.15***	1.34±0.18***	1.38±0.11***
**WHR**	0.73±0.04	0.87±0.08***	0.84±0.08***	0.90±0.06***
**FEV_1_(%pred)**	95.31±10.74	83.52±11.92**	85.78±11.49	81.50±11.12*
**FVC (%pred)**	89.15±10.20	82.16±10.44	84.44±12.20	80.10±8.70
**FEV_1_/FVC (%pred)**	105.9±7.21	102.10±8.67	104.8±8.48	99.70±8.54
**MIP (cmH_2_O)**	81.14±17.39	84.05±32.45	99.50±33.68	71.70±26.93
**MEP (cmH_2_O)**	74.29±19.87	96.94±22.87	102.9±22.49	92.20±23.19

BMI =  body mass index; WC =  waist circumference; HC =  hip circumference; WHR =  waist-to-hip ratio; FEV_1_ =  forced expiratory volume in the first second percentage of predicted value; FVC =  forced vital capacity percentage of predicted value; FEV_1_/FVC =  ratio of forced expiratory volume in the first second and forced vital capacity percentage of predicted value; MIP =  maximal inspiratory pressure; MEP =  maximal expiratory pressure. Data are expressed as mean ± standard deviation. *p<0.05, **p<0.01, ***p<0.001 (vs control); ^°^p<0.05 (vs group 1).

**Figure 2:**
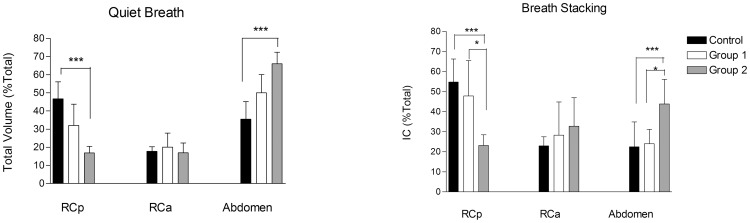
Average tidal volume of the three chest wall compartments (RCp =  pulmonary rib cage; RCa = abdominal rib cage; and AB = abdomen), expressed as percentage of total, during spontaneous quiet breathing (Left panel). Average inspiratory capacity of the three chest wall compartments (RCp =  pulmonary rib cage; RCa = abdominal rib cage; and AB = abdomen) expressed as percentage of total, as assessed during the breath stacking maneuver (Right panel). Black bars:control, white bars: group 1 and Gray bars: group 2. * p<0.05, ***p<0.001.

No significant differences in the ventilatory pattern were also found between groups during quiet breathing in terms of tidal volume, inspiratory and expiratory time and breathing frequency. Minute ventilation in obese was significantly higher than in controls. When considering the two obese groups, minute ventilation was higher only in group 2 compared to controls (p = 0.001). Compartmental volume variations, both expressed in liters (Table 2) and as percentages of total tidal volume (Fig. 2), were not significantly different between the obese groups. Healthy subjects, however, had significantly higher pulmonary rib cage and lower abdominal tidal volume than obese both in liters (p = 0.006 and p<0.001, respectively) and as percentage of tidal volume (p<0.001). Again, when considering the two obese groups, compartmental tidal volumes were significantly different only in group 2 compared to controls (Table 2).

**Table 2:** Ventilatory and chest wall pattern data of control, obese (overall), groups 1 and 2.

	Control	Obese (overall)	Obese (Group 1)	Obese (Group 2)
**N. of subjects**	15	19	9	10
**V_T_ (L)**	0.51±0.20	0.59±0.21	0.52±0.22	0.64±0.20
**Frequency (min^−1^)**	15.10±4.72	18.32±4.79	18.38±4.64	18.27±5.17
**Min. Vent. (L/min)**	7.12±1.54	9.59±2.17***	8.51±1.94	10.57±1.97***
**T_I_ (sec)**	1.75±0.53	1.53±0.44	1.56±0.52	1.51±0.39
**T_E_ (sec)**	2.65±0.95	2.12±0.75	2.07±0.60	2.18±0.89
**V_T,RCp_ (L)**	0.24±0.11	0.14±0.08**	0.17±0.09	0.12±0.06**
**V_T,RCa_ (L)**	0.09±0.04	0.11±0.06	0.10±0.07	0.11±0.05
**V_T,AB_ (L)**	0.18±0.07	0.34±0.13***	0.25±0.09	0.42±0.11***
**IC (L)**	2.55±0.55	2.33±0.43	2.41±0.53	2.24±0.28
**IC_CW_ (L)**	2.03±0.39	2.24±0.75	2.22±0.96	2.59±0.47
**IC_RCp_ (L)**	1.11±0.30	0.76±0.26**	0.94±0.22	0.60±0.17***,^°°°^
**IC_RCa_ (L)**	0.46±0.12	0.80±0.52*	0.76±0.67	0.85±0.38*
**IC_AB_ (L)**	0.46±0.27	0.84±0.46*	0.52±0.26^c^	1.14±0.41***,^°°°^

V_T_ =  tidal volume; Min. Vent. =  minute ventilation; T_I_  =  inspiratory time; T_E_ =  expiratory time; V_T,RCp_, V_T,RCa_ and V_T,AB_  =  percentage contribution of pulmonary rib cage, abdominal rib cage and abdomen to tidal volume; IC,_CW_, IC_,RCp_, IC_,RCa_ and IC,_AB_ =  inspiratory capacity of total chest wall, pulmonary rib cage, abdominal rib cage and abdomen (measured by OEP during breath stacking). Data are expressed as mean ± standard deviation. *p<0.05, **p<0.01, ***p<0.001 (vs control); ^°°°^p<0.001(vs group 1).

During breath stacking, IC measured by the Wright respirometer was not statistically different from inspiratory capacity of the total chest wall (IC_CW_) measured by OEP (Table 2). Both IC and IC_CW_ measured during breath stacking were similar between the three groups (Table 2).

Significant differences were found, however, in the subdivision of IC_CW_ into the three compartments. Obese subjects showed a smaller expansion of the pulmonary rib cage and a greater expansion of the abdomen (p<0.001) compared to controls (Table 2). IC_RCp_ and IC_AB_ were significantly different also between groups 1 and 2. As a consequence, the contributions of the pulmonary rib cage and abdomen to inspiratory capacity were significantly different between the two groups (Figure 2).

**Figure 3:**
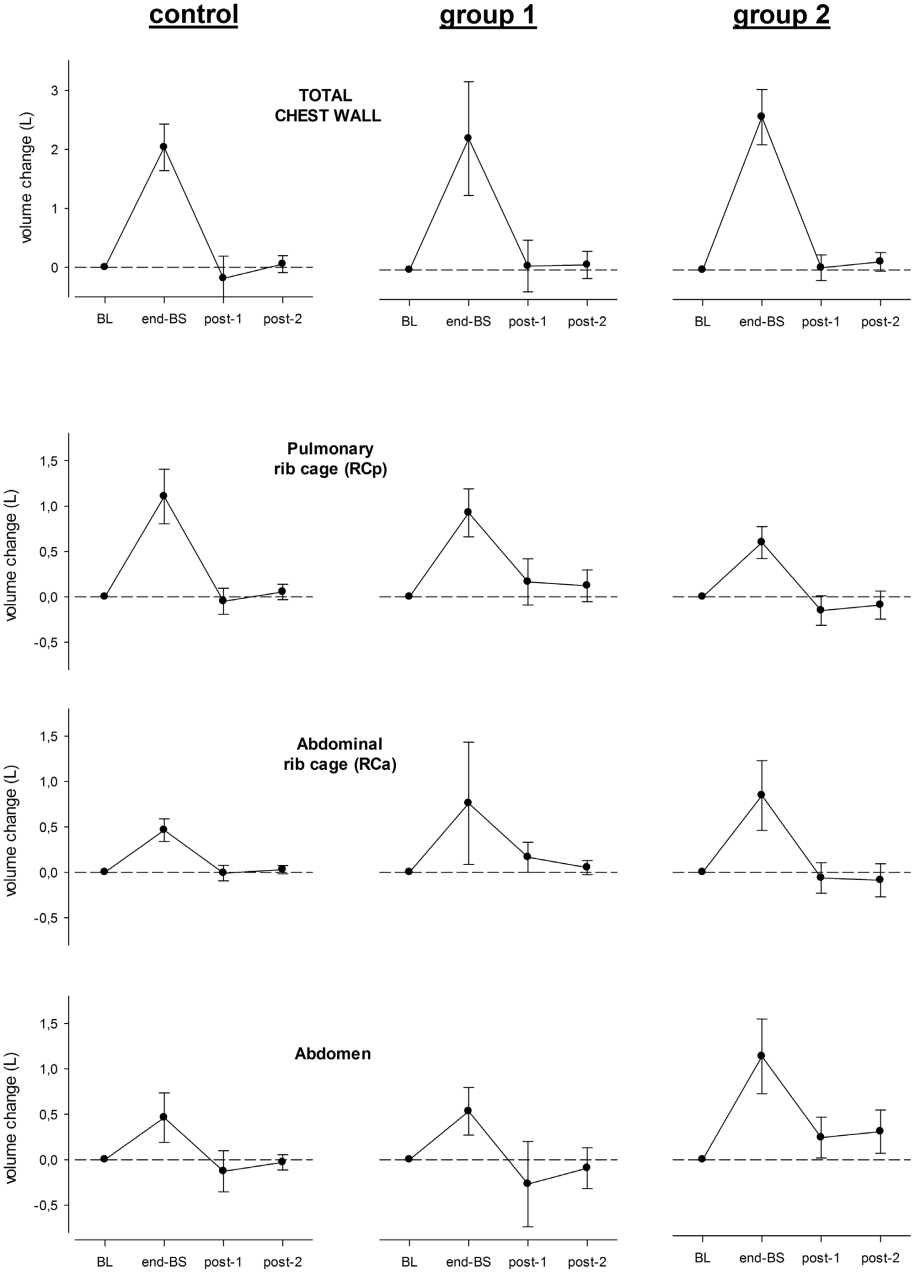
Average variations of total chest wall (Vcw), pulmonary rib cage (Vrc,p), abdominal rib cage (Vrc,a) and abdominal (Vab) volume in control group (left panel, n = 15), group 1 (middle panels, n = 9) and group 2 (right panels, n = 10) at end of breath stacking, i.e. TLC (end-BS), first expiration after breath stacking (post-1) and 30 seconds after breath stacking (post-2) compared to baseline period (BL), (from top to bottom) (see text for details). Data are expressed as mean (SD).

The acute effects of the breath stacking maneuver on V_CW_, V_RC,P_, V_RC,A_ and V_AB_ are shown in Fig. 1 (in form of two representative examples, one of a subject belonging to group 1 and another to group 2) and in Fig. 3 (in terms of average values of total and compartmental volumes at P1, P2, P3 and P4). As described above, the total inspired volume at the end of the breath stacking maneuver was similar between the two groups, with the pulmonary rib cage being the prevalent compartment in group 1 and the abdomen in group 2. After the maneuver, the end-expiratory volume of the total chest wall returned to the baseline value in both groups. In group 1, however, the end-expiratory volume of the pulmonary rib cage tended to remain higher than baseline until P4 (with the abdominal rib cage and the abdomen returning to baseline levels or even to lower values). Conversely, in group 2 the end-expiratory volume of the abdomen remained higher than baseline until P4 (with the two rib cage compartments returning to baseline levels or even to lower values) (Fig. 3).

**Figure 4:**
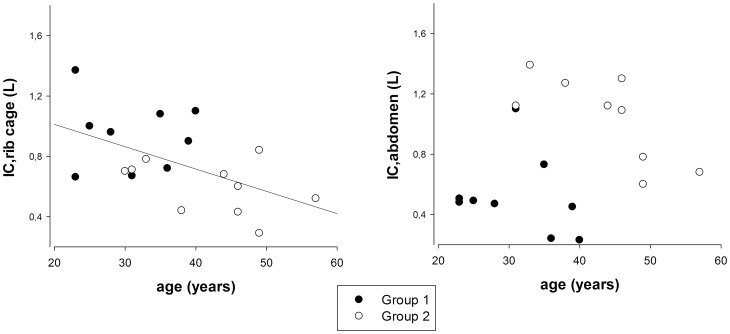
Relationship between inspiratory capacity of pulmonary rib cage (IC,_RCp_)and age (left panel) and between inspiratory capacity of the abdomen and age (right panel). Closed symbols: individuals belonging to group 1. Open symbols: individuals belonging to group 2. The straight line in the left panel shows the linear regression of the data (IC,_RCp_ = 1.308–0.0148 * age, r^2^ = 0.290, p = 0.0017).

A significant inverse linear relationship was found between age and inspiratory capacity of the pulmonary rib cage (IC,_RCp_ = 1.308–0.0148 * age, r^2^ = 0.290, p = 0.0017) but not of the abdomen (Fig. 4).

## Discussion

The main result of the present study is that obese women demonstrate different responses in the different compartments of the chest wall during breath stacking maneuver. During the maneuver, the younger obese women expand the pulmonary rib cage similarly to healthy controls, while older obese women showed a restricted pattern in this compartment. In other terms, our results show that in obese women rib cage mobility decreases with age. This is demonstrated by a) the significant different age and inspiratory capacity of the pulmonary rib cage as assessed during breath stacking between group 1 and 2 (Table 2) and b) the significant inverse linear relationship between age and inspiratory capacity of the pulmonary rib cage (Fig. 4).

It has been reported that in healthy female subjects, the maximal maturation of the respiratory system occurs at about 20 years. Lung function remains stable with very minimal change from 20 to 35 years and starts declining very slowly thereafter, due to the decrease in chest wall compliance, lung static recoil and respiratory muscles strength [26]–[28]. In obese patients, the decline is presumably accelerated. This was evident in our population, particularly in terms of maximal inspiratory pressure and rib cage mobility. Subjects who responded to the breath stacking by expanding the pulmonary rib cage more than the abdomen were younger compared to those who responded by expanding more the abdomen. Our results suggest therefore that ageing influence in obese women the expansion of the chest wall in terms of decreased compliance of the rib cage and strength of inspiratory muscles. In addition, we found that in older women the displacement of the abdomen is augmented during breath stacking. Thus, it can be assumed that this maneuver may facilitate diaphragmatic motion. This may have important effects on ventilation in the more basal regions of the lung. In a previous study from our group [29] based on scintigraphic measurements with inhaled radioaerosol, it was shown that in healthy individuals breath stacking allows more deposition of the radiopharmaceutical in the peripheral regions and in the lower third of the lung compared to incentive spirometry that instead caused greater deposition in the central regions and in the middle third of the lung.

Regarding lung function and ventilatory pattern, we did not find any difference between younger and older obese women. This was probably due to the fact that the two groups were similar in terms of BMI and other anthropometric parameters. It is important to note, however, that obese women present an altered lung and thoraco-abdominal function, characterized by a restrictive pattern, and abnormal thoraco-abdominal motion during spontaneous breathing compared to normal weight women, as shown by the comparison between the overall obese groups and controls in the present study, that confirms recent findings [18]. In obese women the largest contribution observed in the abdominal compartment during quiet breathing was different from that found in a study involving healthy female subjects with normal weight, where the prevalent contribution to tidal volume was instead in the two rib cage compartments [30]. It can be assumed that the predominant contribution of abdominal compartment is due to greater variation in tidal volume caused by the diaphragm and abdominal muscles in an attempt to offset the burden caused by the accumulation of fat in chest and abdomen [18].

Obese patients are particularly vulnerable to surgery that often causes deterioration in lung function due to factors such as anesthesia, supine positioning, duration of surgery, pain, and often results in a rapid and shallow breathing pattern [11], [9]. Breath stacking maneuver, therefore, appears to be a possible therapy for preventing these complications, especially for those patients who present difficulty of generating high volumes and sustain the inspired volume. This is supported by the recent work of Dias et al. [31], who studied patients undergoing abdominal surgery, and compared breath stacking with incentive spirometry. These authors found that the decrease in lung volume after surgery was smaller in the patients treated with breath stacking and that this maneuver allowed greater lung expansion and greater maintenance of inspiratory volume. Breath stacking has also been proposed, in a modified form using a manual resuscitation bag, a one-way valve and a mask or mouthpiece, to increase the maximum inspiratory capacity and expiratory flow to improve cough in patients with neuromuscular disorders or spontaneously breathing intubated [32]–[34].

A possible limitation of this study is that rib cage compliance was not directly measured. This would have implied the use of an esophageal balloon-catheter system that is scarcely tolerated by the subjects and can be considered an invasive procedure. It should be noted that we designed our study in order to have only noninvasive procedures and techniques. No volunteer had an adverse effect arising from the technique. Only two women reported fear of getting suffocated by the mask, a problem that was solved with detailed explanations of the procedure and prior training maneuvers.

Another possible limitation is the use of OEP, a measurement technique that has been rarely used in obese women in the past. We have recently shown, however, that in obese women during quiet breathing there is a good agreement between V_CW_ variations measured by OEP and lung volume variations measured by integrating airflow measured at the mouth by a pneumotachograph [18]. In the present paper we now also show that IC values measured by the Wright respirometer are in a good agreement with IC_CW_ values measured by OEP. It must be noted that IC tended to be slightly lower than IC_CW_, probably due to gas expansion occurring at high lung volumes.

In conclusion, our results show that not only the spontaneous breathing, but also the maximal expansion of the chest in the different thoraco-abdominal compartments are influenced by obesity in women. The expansion of rib cage and abdomen during breath stacking maneuver is influenced by age, and not by any anthropometric parameter. This should be confirmed in future studies considering larger populations of obese subjects with different age and in situations like post-bariatric surgery, which is now commonly used in the clinical practice as a treatment for obesity.
